# Social Beliefs and Visual Attention: How the Social Relevance of a Cue Influences Spatial Orienting

**DOI:** 10.1111/cogs.12529

**Published:** 2017-11-02

**Authors:** Matthias S. Gobel, Miles R. A. Tufft, Daniel C. Richardson

**Affiliations:** ^1^ SAGE Center for the Study of the Mind Department of Psychological and Brain Sciences University of California at Santa Barbara; ^2^ Department of Experimental Psychology University College London

**Keywords:** Attention, Inhibition of return, Joint action, Social cognition, Social status, Spatial cueing

## Abstract

We are highly tuned to each other's visual attention. Perceiving the eye or hand movements of another person can influence the timing of a saccade or the reach of our own. However, the explanation for such spatial orienting in interpersonal contexts remains disputed. Is it due to the *social appearance* of the cue—a hand or an eye—or due to its *social relevance*—a cue that is connected to another person with attentional and intentional states? We developed an interpersonal version of the Posner spatial cueing paradigm. Participants saw a cue and detected a target at the same or a different location, while interacting with an unseen partner. Participants were led to believe that the cue was either connected to the gaze location of their partner or was generated randomly by a computer (Experiment 1), and that their partner had higher or lower social rank while engaged in the same or a different task (Experiment 2). We found that spatial cue‐target compatibility effects were greater when the cue related to a partner's gaze. This effect was amplified by the partner's social rank, but only when participants believed their partner was engaged in the same task. Taken together, this is strong evidence in support of the idea that spatial orienting is interpersonally attuned to the *social relevance* of the cue—whether the cue is connected to another person, who this person is, and what this person is doing—and does not exclusively rely on the social appearance of the cue. Visual attention is not only guided by the physical salience of one's environment but also by the mental representation of its social relevance.

## 1 Introduction

Our eyes are in constant demand: Turn signals, pointed glances, and flashing banner ads all clamor for our visual attention. Some of these cues—glances, head turns, pointing fingers—are generated by other people. Others—warning lights, traffic signals, and signposts—are put there intentionally by other people as a signal. And others still—the bright plumage of a bird, a dark rain cloud, or paw prints in the mud—are oblivious to our presence.

Does the visual attention system respond equally to all these cues? Specifically, do we give special weight to locations or objects in the world that are cued by other people since they might indicate their mental states or communicative intentions? Further, if spatial orienting is socially attuned, is this a response to the *social appearance* of the cue (e.g., facial features or a finger) or a response to its *social relevance*, the fact that the cue is connected to another intentional being with her own states of attention?

Here, we address the question of social relevance by spatially cueing participants' attention while manipulating their beliefs about the social or non‐social origins of those cues. In this way, we are able to investigate how changes in the social relevance of the cue—whether the cue is connected to another person, who that person is, and what that person is doing—influence spatial orienting. We make use of a robust feature of spatial attention and place it in a social context that can be experimentally manipulated.

### 1.1 Social attention

Since the very first eye tracking experiments, it has been shown that people pay increased attention to the eyes of others (Yarbus, 1965). Moreover, they focus their attention to where other people are looking (Frischen, Bayliss, & Tipper, 2007). Humans are able to use each other's eye movements to efficiently cooperate and communicate with each other. For example, attending to the focus of another's attention can tell us where reward or danger is lurking in the environment. Indeed, this “social attention” has a key function in various aspects of social life facilitating interpersonal communication, successful cooperation and human interdependence (Klein, Shepherd, & Platt, 2009; Richardson & Gobel, 2015; Risko, Richardson, & Kingstone, 2016).

For example, in gaze‐cuing paradigms, it has been shown that the visual attention of others can direct our own attention. In such experiments, participants are typically shown a centrally presented drawing or picture of a face gazing in a certain direction, and they are instructed to respond as quickly as possible to targets appearing either congruent with gaze direction or incongruent (e.g., Friesen & Kingstone, 1998). Despite gaze being non‐predictive of targets, it has repeatedly been shown that reaction times to congruently cued targets are facilitated (Frischen et al., 2007).

In these experiments on “social attention” (reviewed in Richardson & Gobel, 2015), participants are usually presented with unambiguous, visibly social cues, such as faces. So it is not clear whether shifts in attention are triggered by the *social appearance* of the cues or by the *belief* that the cues are connected to another person. Indeed, while some researchers found that mental state attributions modulated gaze cueing effects (Nuku & Bekkering, 2008; Teufel, Alexis, Clayton, & Davis, 2010a; Teufel, Fletcher, & Davis, 2010b; Wiese, Wykowska, Zwickel, Mu, & Müller, 2012), others have failed to find the same (Cole, Smith, & Atkinson, 2015; Quadflieg, Mason, & Macrae, 2004). Thus, the empirical literature remains inconsistent on the role that beliefs about the social relevance of gaze cues play in the allocation of visual attention.

Here, we go beyond the previous literature and ask, what are the minimal conditions for social context to influence visual attention? Most researchers maintain that people use prior knowledge and current goals to selectively orient their attention to non‐social stimuli (Bundesen, 1990; Desimone & Duncan, 1995; Folk, Remington, & Johnston, 1992). So would beliefs about the social origin of stimuli that are non‐social in appearance, such as a dot representing the gaze of another person, suffice to influence basic attention? In order to answer this question, we turned away from gaze cueing in which a social stimulus, that is, a face, is presented, and we utilized another widely used paradigm in attention research: spatial orienting.

### 1.2 Interpersonally attuned spatial orienting

Our central question is whether aspects of basic attention, in this case spatial orienting, can be influenced by beliefs about the social relevance of a visual cue—whether the cue is connected to another person, who that person is, and what that person is doing—even when the stimuli are non‐social in nature. In spatial orienting paradigms, participants' visual attention is cued to one spatial location, and they are subsequently asked to respond to the same or a different spatial location. A substantial literature shows that people are slower to return their attention back to a location that it has previously occupied (e.g., Dukewich & Klein, 2015; Klein, 2000); this is called the inhibition of return (IOR) effect (Posner, Rafal, Choate, & Vaughan, 1985). It is argued that IOR might play an adaptive role promoting efficient visual search by biasing attention toward novelty and away from previously attended objects or locations (Klein & MacInnes, 1999).

There are good reasons to think that it would be adaptive for spatial orienting to be responsive to the social relevance of another person. If it is true that IOR makes search more efficient for an individual, then it could also make search more efficient for people working together. Individuals who are engaged in joint action (Knoblich & Sebanz, 2006; Sebanz, Bekkering, & Knoblich, 2006) or joint perception (Richardson et al., 2012) are tuned to the cognitive representations and locus of their partner's attention. Brennan, Chen, Dickinson, Neider, and Zelinsky (2008), for example, found that two individuals performing a visual search task were highly efficient when they could see each other's gaze location.

Indeed, previous work has documented interpersonally attuned spatial orienting between two people, in which one participant is slower to reach to locations that were previously touched by another participant who sat opposite (Welsh et al., 2005). Some see this as evidence that the participants are representing each other's actions through a “mirror system” (Rizzolatti & Craighero, 2004) that facilitates the imitation of their movements (Ondobaka, de Lange, Newman‐Norlund, Wiemers, & Bekkering, 2012; Ondobaka, Newman‐Norlund, De Lange, & Bekkering, 2013) and may even respond to the specific goals of other people (Hayes, Hansen, & Elliott, 2010; Welsh et al., 2005, 2007).

However, it is debated whether such interpersonal spatial orienting effects are really “social” at all. Other work suggests it is simply the participants' reaching movements that are serving as a visual cue and manipulating their spatial orienting, much as any other stimulus would (Atkinson, Simpson, Skarratt, & Cole, 2014; Cole, Skarratt, & Billing, 2012; Doneva, Atkinson, Skarratt, & Cole, 2015; Skarratt, Cole, & Kuhn, 2012). According to this view, spatial orienting between two people can be accounted for by mechanisms that do not require any mental representation of who the other person is and what she is doing, because it is simply an example of a standard orienting effect where the cue happens to be a hand.

On the other hand, if spatial orienting between two people is influenced by nuances of social relevance, then this would be strong evidence for truly interpersonally attuned spatial orienting and for social cognition that can guide visual attention even at basic levels, when the stimuli are non‐social in nature.

### 1.3 The current research

The purpose of our research is to test whether the social relevance of a visual cue—whether the cue is connected to another person, who that person is, and what that person is doing—influences spatial orienting effects. The answer to this question would reveal the scope of social effects on basic visual attention and contribute to the debate about whether “reaching social IOR effects” are really social at all. To this end, we developed an interpersonal version of the spatial cueing paradigm (Posner et al., 1985). The classic spatial orienting effect is that participants are slower to respond to a target when it appears in the same location as a preceding cue (Posner, Snyder, & Davidson, 1980). In our experiments, pairs of participants were seated in the same room but did not see each other or interact during the experiment. In fact, none of the stimuli they saw had an explicit, visible social content. Any difference in participants' spatial orienting would show that beliefs about the social relevance of the cue changes how attention is deployed, regardless of the cue's appearance.

Experiments 1 tests whether believing that an onscreen cue relates to another person moderates spatial orienting. Experiment 2 tests whether beliefs about who that other person is and whether that other person is task relevant (engaged in the same or a different task) moderates interpersonal spatial orienting. In all our experiments, every participant sees exactly the same visual stimuli. The only thing manipulated is the participant's belief about the nature of the interaction with the other person; therefore, any changes in spatial orienting would provide strong evidence for a truly interpersonally attuned visual system and social cognition that penetrates basic spatial orienting independent of the social appearance of the stimulus.

## 2 Experiment 1

Our goal is to demonstrate that the social relevance of a cue—whether or not participants believe it relates to another person—will modulate spatial orienting. We were inspired by previous research that presented another person as an explicitly social stimulus (e.g., Skarratt, Cole, & Kingstone, 2010; Welsh et al., 2005). Perceiving the actions of another person can slow responses to a target location in exactly the same way as a participant's own reach or gaze shift. In our experiment, however, participants did not see another person: We simply manipulated their belief about whether a disembodied cue had social relevance, reflecting the gaze position of the interaction partner, or not.

### 2.1 Method

#### 2.1.1 Participants

Here, and in the subsequent experiment, we estimated sample sizes based on previous research on interpersonal IOR, showing moderate to large effect sizes, and targeted to sample at least 45 subjects in each experiment in order to have 80% power for detection of a medium‐sized effect when employing the traditional 0.05 criterion for statistical significance. Ethical approval for all experiments was provided by the UCL Experimental Psychology department.

Sixty‐three participants volunteered to participate in exchange for payment and performed the experiment with a confederate. The confederate was one of four undergraduate research assistants (2 female and 2 male), which were randomly assigned to participants, so that none of the observed effects would be due to any specific identity of the confederate. 14 participants were excluded because they were suspicious about the identity of the confederate or failed to complete the task. Thus, we analyzed data from 49 participants (29 females, *M*_age_ = 26.12, *SD*_age_ = 8.51).

#### 2.1.2 Design

We employed a 2 (Target Location: cued vs. uncued) × 2 (Cue Origin: human‐generated vs. computer‐generated) × 2 (Background Pictures: present vs. absent) mixed‐factor design, where the dependent variable was participants' reaction times to the onset of the target stimulus. Because the participants' gender did not moderate the effects reported below, it was not included as factor in our analyses.

#### 2.1.3 Apparatus

The participant and the confederate arrived at the same time and were then seated in opposing corners of the experimental 25 m^2^ room, such that they had their backs to each other and could not see each other's screens or actions. We told them to not talk or interact during the experimental task. Participants faced a 19″ LCD screen positioned at a distance of approximately 60 cm. A custom‐built remote eye tracker was positioned at the base of the screen. The participant and the confederate were both provided with a wireless computer mouse to record their responses.

#### 2.1.4 Procedure

We told the participant and the confederate that both of them would see the same set of pictures (see below for rationale of including pictures) or the same screen with four white quadrants and a series of geometrical symbols. For both of them, their job was to respond as quickly as possible to the appearance of a blue square. We explained to both that eye trackers would monitor their eye movements during the task and that at times these eye trackers could be connected so that they would see where each other had just looked at. To bolster the critical manipulation of our study, the participant and the confederate were both taken through an eye tracker calibration sequence at the beginning of the experiment in order to make participants believe that the eye tracker worked, could be connected, and would show where each other was looking on screen.

We informed the participant and the confederate that throughout the experiment a red dot would appear, and that half of the times this red dot represented where their partner had preferentially looked at on their screen (human‐generated cue trials), whereas the other half of times the red dot's location would be randomly chosen by the computer (computer‐generated cue trials). To signal whether participants were about to engage in human‐ or computer‐generated cue trials, before the start of every block, a message appeared on the screen indicating whether the eye trackers were “connected” or “not connected.” The red dot was, of course, always computer‐generated. Participants never received any information from their partner and always carried out the spatial cueing task independently. The confederate never actively participated in the experiment but remained silently seated back to back with the participant.

**Figure 1:**
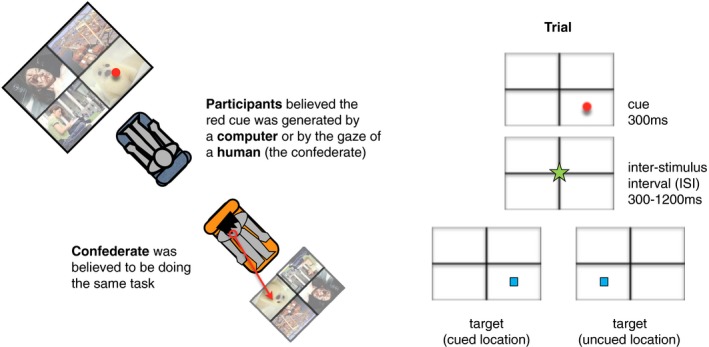
Schema of the Experiment. We asked participants to perform a spatial cueing paradigm with another person (a confederate). Participants believed that the red cue represented a location chosen at random by the computer or their partner's gaze location. The condition with pictures present is depicted here (left); for half of the participants only an empty grid was shown (right).

The trial design is shown in Fig. 1. Participants saw a 2 × 2 grid that for half of the participants presented for 1,200 ms four pictures taken at random from the IAPS normed database (Lang, Bradley, & Cuthbert, 2005), whereas for the other half of participants the grid remained empty. To make it plausible as to why the confederate might be looking at one region rather than another, for half of the participants, we decided to show background pictures on screen. Yet, to remove the possibility that in the social context condition, the participants were explicitly imagining what the confederate was thinking while looking at each picture, we removed the pictures for the second half of participants. In fact, the presence or absence of background pictures did not change results, as reported below.

Next, a red dot appeared in the same locations of one of the four quadrants for 300 ms (visual cue). In the human‐generated cue condition, participants believed that the cue represented where the interaction partner had preferentially looked at; and in the computer‐generated cue condition that it was chosen at random by the computer. After the cue disappeared, a green spinning star appeared centrally for between 300 and 1,200 ms (intervening cue), followed by a blue square (visual target), which appeared at either the cued or uncued location, and participants responded to it as fast as possible. The varying interstimulus interval was simply used as jitter so that the trial onsets were not predictable, as we had no theoretical predictions about the potential time course of the effects of social information.

Participants completed a total of 288 trials during a single session, of which 24 were catch trials where no blue square was presented. The remaining trials were randomly split between four SOAs (600 ms, 900 ms, 1,200 ms, 1,500 ms), which fall within the range of SOAs that have been shown to reliably illicit IOR effects (e.g., Klein, 2000). Twenty‐five percent of all trials were cued trials and 75% of all trials were uncued trials. A testing session consisted of four blocks (two social and two non‐social), with the order of presentation counterbalanced across participants. Participants pressed a mouse button with their dominant (writing) hand as quickly as possible on seeing the blue square (target detection task). If no response was given after 3,000 ms, the trial ended and the next trial began.

### 2.2 Results

We analyzed data from trials where cues and targets appeared on screen. We excluded trials (0.90% of the data) where participants anticipated (RT < 100 ms) or failed to respond (RT > 3,000 ms). We chose to analyze the data in two complementary ways.

In a first step, we conducted a three‐factorial mixed‐design Analysis of Variance on participants' mean reaction times and entered Background Pictures as a between‐subject factor and Target Location as well as Cue Origin as within‐subject factors. This three‐factorial mixed‐design anova revealed a significant main effect of Background Pictures, *F*(1, 47) = 10.48, *p* = .002, $\eta_{p}^{2}$ = .18, such that participants were overall slower in trials with background pictures (*M = *470 ms, *SEM* = 15 ms) than when background pictures were absent (*M = *402 ms, *SEM* = 15 ms), suggesting that an increase in visual information increased participants' response times. We also observed a significant main effect of Target Location, with cued trials (*M = *445 ms, *SEM* = 11 ms) slower than uncued trials (*M = *427 ms, *SEM* = 10 ms), consistent with an overall IOR effect of about 17 ms, *F*(1, 47) = 29.97, *p *<* *.001, $\eta_{p}^{2}$ = .39. There was also a main effect of Cue Origin, *F*(1, 47) = 15.20, *p *<* *.001, $\eta_{p}^{2}$ = .24, with participants significantly slower overall on human‐generated cue trials (*M* = 448 ms, *SEM* = 12 ms) compared to computer‐generated cue trials (*M = *424 ms, *SEM* = 10 ms), suggesting that an increase in social information increased participants' response times.

Importantly, these effects were qualified by a significant interaction effect between Target Location and Cue Origin, *F*(1, 47) = 5.02, *p = *.03, $\eta_{p}^{2}$ = .10. In human‐generated cue trials, participants were significantly slower to respond to cued locations (*M = *460 ms, *SEM* = 13 ms) than uncued locations (*M = *437 ms, *SEM* = 12 ms), *p *<* *.001. This difference was reduced for computer‐generated cue trials (*M*_cued_ = 430 ms, *SEM*_cued_ = 10 ms; and *M*_uncued_ = 418 ms, *SEM*_uncued_ = 10 ms, respectively; *p = *.002). In other words, the IOR magnitude on human‐generated cue trials (*M = *23 ms, *SEM* = 5 ms) was significantly greater than on computer‐generated cue trials (*M = *12 ms, *SEM* = 4 ms), *t*(48) = 2.23, *p = *.03, *d = *0.64.

We reran our analyses with SOA as an additional within‐subject factor. The four‐way interaction of SOA by background pictures by target location by cue origin did not reach conventional levels of significance, *F*(3, 141) < 1, *p = *.71, $\eta_{p}^{2}$ = .01, and neither did the three‐way interaction of SOA by target location by cue origin, *F*(3, 141) = 1.99, *p = *.12, $\eta_{p}^{2}$ = .04, or any other interactions with SOA, *F*s < 2. Table 1 shows means and standard errors across social contexts and different SOA levels.

**Table 1:** Mean RTs in ms (with *SE*) for Experiment 1

	Experimental Condition			
	Human‐Generated		Computer‐Generated	
SOA	*Cued*	*Uncued*	*Cued*	*Uncued*
600 ms	484 (16)	476 (14)	464 (12)	444 (11)
900 ms	462 (13)	425 (11)	431 (12)	412 (11)
1,200 ms	450 (15)	422 (12)	421 (13)	415 (11)
1,500 ms	446 (13)	425 (12)	415 (11)	403 (10)
Average	460 (13)	437 (12)	430 (10)	418 (10)

In a second step, we employed a Bayesian analysis of our results, since in addition to avoiding some of the problems of null hypothesis significance testing, these analyses are able to estimate the relative strength of evidence for and against null and alternative hypotheses (Dienes, 2011; Kruschke, 2010, 2011; Wagenmakers, Wetzels, Borsboom, & van der Maas, 2011). For each subject, within the human‐generated cue and computer‐generated cue conditions, we first calculated the size of the IOR effect by subtracting the mean RT of uncued trials from the cued trials.

**Figure 2:**
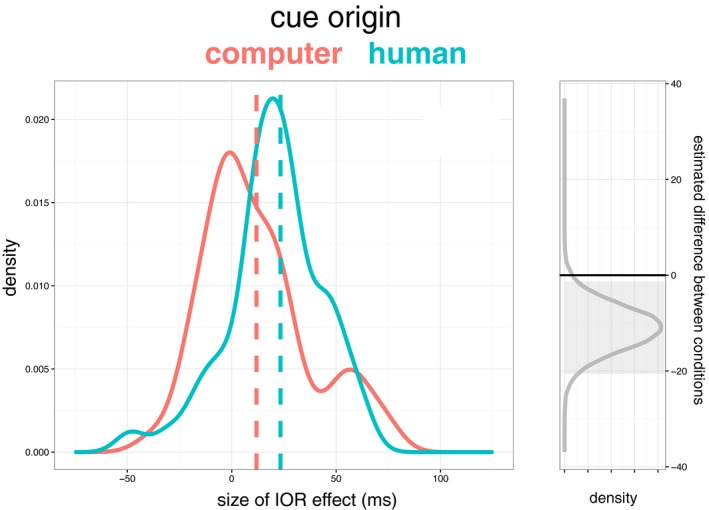
Red and blue lines show the distribution of inhibition of return effects for each subject in each condition, with dashed lines indicating the means for each condition. On the right, the gray line shows the posterior probability distributions of the difference between conditions, and the gray area shows the 95% credibility interval (HDI) for this difference.

Fig. 2 shows a density plot of these participant IOR effects for the two cue origin conditions. Following Kruschke (2010, 2011, 2013) and using the BEST package for R (Kruschke & Meredith, 2014), we generated posterior probability distributions to compare the means of the two conditions. The right plot of Fig. 2 shows the distribution of estimates for the difference between conditions. The 95% Bayesian credibility interval, shown by the gray box, is from −20.6 to −1.3, showing that there is good evidence for a non‐zero difference between the IOR effects in each condition. Importantly, we find converging evidence that believing a non‐social cue represents another person's gaze location modulates IOR effects.

### 2.3 Discussion

Experiment 1 provides initial evidence for the idea that the social relevance of a cue influences spatial orienting. Manipulating the *beliefs* about the social origin of non‐social cues alone was sufficient to significantly amplify the IOR magnitude. Since no sensory characteristics of the stimuli were changed across conditions, IOR differences when the cue origin was a computer compared to when the cue origin was another human cannot be attributed to the physical appearance of the stimuli.

## 3 Experiment 2

Experiment 2 aimed to provide further insight into how the social relevance of another person impacts interpersonal spatial orienting effects. First, we wanted to understand the depth of the effects on visual attention: Does it matter who is looking? To answer this question, we manipulated beliefs about one of the most critical social dimensions: social hierarchy.

Social hierarchy is one fundamental aspect of social life, structuring interactions in families, teams, and entire societies (Fiske, 2010). Evolutionary scholars suggest that hierarchies have been important across our history, because they coordinate group members' actions, from hunting prey to organizing labor, improving the group's performance overall (e.g., King, Johnson, & Van Vugt, 2009; Van Vugt, 2006). The general idea is that groups award the highest social rank to the individual that they believe to best represent their interests. By choosing the best candidate to lead, the group should do better overall, and individual group members would increase their chances of survival and reproduction.

There are many ways in which organizing groups hierarchically can be beneficial. For example, social hierarchy distributes resources and thereby reduces intragroup conflict (Anderson, Srivastava, Beer, Spataro, & Chatman, 2006; Bendersky & Hays, 2012). Moreover, social hierarchy leads to more efficient decision making (Van Vugt, Hogan, & Kaiser, 2008), coordinates collective locomotion (Blau, 1964; Hardy & Van Vugt, 2006; Keltner, Van Kleef, Chen, & Kraus, 2008; Thibaut & Kelley, 1959; Willer, 2009), and increases team performance (Anicich, Swaab, & Galinsky, 2015; Halevy, Chou, Galinsky, & Murnighan, 2012; Ronay, Greenaway, Anicich, & Galinsky, 2012). In contrast, if there is no clear hierarchical structure, group performance suffers (Bendersky & Hays, 2012; Greer, Caruso, & Jehn, 2011; Greer & van Kleef, 2010). Thus, social hierarchy has a key function in coordinating social living in groups.

One potential mechanism through which behavioral coordination in groups is accomplished is the way group members' visual attention is guided by higher ranked individuals. Indeed, past research has shown that both gazing at and gazing with others are modulated by targets' social rank. People are more likely to look at higher rather than at lower ranked individuals (Cheng, Tracy, Foulsham, Kingstone, & Henrich, 2013), especially their eyes (Foulsham, Cheng, Tracy, Henrich, & Kingstone, 2010), and are more likely to follow their gaze (Dalmaso, Pavan, Castelli, & Galfano, 2012). Therefore, we reasoned that whether a person of higher or a person of lower rank was believed to be gazing with the participant would modulate interpersonal spatial orienting effects, as it provided critical information about who the interaction partner would be.

Second, we wanted to understand the breadth of the effects on visual attention: Does it matter what counts as looking “together”? To answer the question, we dissociated two alternatives: Would changes in spatial orienting occur when two people believed that they were looking at the same stimuli at the same time or would they also need to believe that they were doing the same task? Experiment 1 does not allow us to answer this question, since participants always believed they were doing the same task. Thus, in Experiment 2, we manipulated whether participants believed that they were engaged in the same task or a different task to their interaction partner.

Cognitive scientists have argued for a long time that visual attention holds a key function in the coordination of interactions between individuals (Clark, 1996). For example, research in joint action (Sebanz et al., 2006) and joint perception (Richardson et al., 2012) suggests that when people construe a task as shared, they are influenced by each other's cognitive representations. Through eye movements alone, people can actively communicate intentions to one another and successfully cooperate (Brennan et al., 2008). Thus, we reasoned that changes in interpersonal spatial orienting would only occur, when participants believed they were doing the same task as their interaction partner, as it provided critical information about their social relevance.

In Experiment 2, we asked participants to perform a spatial cueing paradigm when gazing with a confederate, who was described as either higher or lower in social rank. Sometimes we told participants they were doing the same task as the confederate but other times that they were doing a different task as the confederate. We contrasted two hypotheses accounting for how social rank would influence interpersonal spatial orienting effects.

The *attentional state hypothesis* predicts that a higher ranked interaction partner orients the onlooker's visual attention automatically, regardless of what the interaction partner is doing. In contrast, the *intentional state hypothesis* holds that a higher ranked interaction partner does not orient visual attention automatically, but only if that person is task relevant. Only when the onlooker shares the same task as a higher ranked interaction partner would the latter guide the onlooker's visual attention.

Evidence in support of the *attentional state hypothesis* will show a modulation of the spatial cue‐target compatibility effects as a function of the interaction partner's social rank independent of whether or not participants believe they are doing the same or a different task as their interaction partner. Evidence in support of the *intentional state hypothesis*, however, will only show a modulation of the spatial cue‐target compatibility effects as a function of the interaction partner's social rank when participants believe they are doing the same task as their interaction partner.

### 3.1 Method

#### 3.1.1 Participants

Forty‐five first‐year undergraduate students (42 females, *M*_age_ = 20.64, *SD*_age_ = 4.29) volunteered in exchange for partial course credit and performed the experiment with a female confederate. In this experiment, we relied on the assistance of the same undergraduate student for the entire data collection. Because the participants' gender did not moderate the effects reported below, it was not included as factor in our analyses. All participants were included in data analysis, as none of them raised suspicion about the true nature of the confederate.

#### 3.1.2 Design

We employed a 2 (Partner Rank: higher rank vs. lower rank) × 2 (Target Location: cued vs. uncued) × 2 (Task Type: same vs. different) mixed‐factor design, where the former factor was manipulated between and the latter two factors were manipulated within subjects. The dependent variable was participants' reaction time to the onset of the target stimulus.

#### 3.1.3 Apparatus

The participant and a confederate arrived at the same time and were then seated in opposing corners of the experimental 25 m^2^ room, such that they had their backs to each other and could not see each other's screens or actions. We told participants to not talk or interact during the experimental task. The confederate was a female undergraduate student, who matched the majority of participants in age, gender, and ethnicity.

#### 3.1.4 Procedure

Participants carried out the interpersonal spatial cueing paradigm as in Experiment 1 along with the confederate. This time, participants were told that the red dot always represented their partner's gaze location. We also explained to them that during half of the experiment they would engage in the same task as their partner, but in the other half of the experiment, they would engage in two different tasks. In the latter case, one of them would continue to respond to the blue square, whereas the other one would memorize the images presented on screen. In reality, participants were always assigned to detect the blue square, that is, to carry out the spatial cueing task. To signal whether participants were about to engage in the same or a different task, a message appeared on screen indicating task type before the start of every block. The order in which participants completed the six experimental blocks (three blocks same task and three blocks different task) was counterbalanced between participants, such that half of the participants started with three same task blocks, whereas the other half of participants started with three different task blocks. The confederate never actively participated in the experiment but remained silently seated back‐to‐back with the participant. Participants completed a total of 432 trials, 48 of which were catch trials during which no blue target was presented. The remaining trials were randomly split between three SOAs (700, 1,100, and 1,500 ms).

#### 3.1.5 Partner rank manipulation

Before participants started the spatial cueing task, we asked them to fill in a brief questionnaire about themselves, including demographic information. We also told participants that they would see some of their partner's answers. Participants were randomly allocated to a higher or lower ranked partner condition. In the higher ranked partner condition, participants read that the interaction partner attended a top‐tier university and came from a very affluent family with parents having prestigious occupations. In the lower ranked partner condition, participants read that their interaction partner attended a bottom‐tier university and came from a less affluent family with parents having less prestigious occupations.

#### 3.1.6 Manipulation check

After participants had completed the spatial cueing task, we asked them to fill in another brief questionnaire. This time, we were interested in their impressions of their interaction partner. Among various filler items, we included the following three items assessing partner's perceived social rank: “*My partner has high social status*.” “*My partner occupies high social rank*.” “*My partner has higher social status than me*.” Participants indicated their agreement with these statements along a 7‐point scale ranging from “*1 = strongly disagree*” to “*7 = strongly agree*.”

### 3.2 Results

#### 3.2.1 Perceived social rank

Our social rank manipulation was successful. Since the three perceived social rank items were highly correlated α = .91, we computed a perceived partner rank score (*M = *4.72, *SD* = 1.13). We submitted this score to an independent‐samples *t* test. As expected, confederates in the high‐rank condition (*N = *24) were perceived to be higher in social rank than confederates in the low‐rank condition (*N = *21) (*M = *5.36, *SD = *1.05, and *M = *3.98, *SD = *0.70, respectively), *t*(40.22)* = *5.25, *p *<* *.001, *d = *1.55. (We corrected degrees of freedom to account for unequal variance between groups.)

#### 3.2.2 Spatial orienting effects

As before, data from trials where cues and targets appeared on screen were analyzed. We excluded trials (1.14% of the data) where participants anticipated (RT < 100 ms) or failed to respond (RT > 3,000 ms).

First, we conducted a three‐factorial mixed‐design Analysis of Variance on participants' mean reaction times, and we entered Partner Rank as a between‐subject factor and Target Location as well as Task Type as within‐subject factors. We then followed up with Bayesian statistics on the IOR effect for each participant in each condition by subtracting the reaction times to uncued trials from cued trials and generating posterior probability distributions for the estimates of condition differences. Across both analyses and consistent with the *intentional state* hypothesis, we found that IOR effects were significantly larger when participants interacted with higher compared to lower ranked confederates, but only if participants believed they were engaged in the same task as the confederate. Since SOA did not interact with our variables of interest, we did not include SOA as factor in subsequent analyses. Mean RTs and standard errors for all experimental conditions and across levels of SOA are presented in Table 2.

**Table 2:** Mean RTs in ms (with *SE*) for Experiment 2

Rank		Task			
		Same Task		Different Task	
	SOA	*Cued*	*Uncued*	*Cued*	*Uncued*
Lower rank partner	700 ms	509 (30)	494 (25)	502 (21)	487 (21)
	1,100 ms	456 (21)	450 (23)	479 (24)	452 (20)
	1,500 ms	452 (28)	438 (22)	451 (21)	447 (19)
	Average	472 (24)	460 (23)	479 (21)	461 (19)
Higher rank partner	700 ms	494 (28)	438 (24)	476 (19)	448 (19)
	1,100 ms	448 (19)	418 (22)	431 (22)	421 (19)
	1,500 ms	460 (27)	424 (20)	432 (19)	416 (17)
	Average	469 (23)	426 (21)	446 (19)	427 (18)

**Figure 3:**
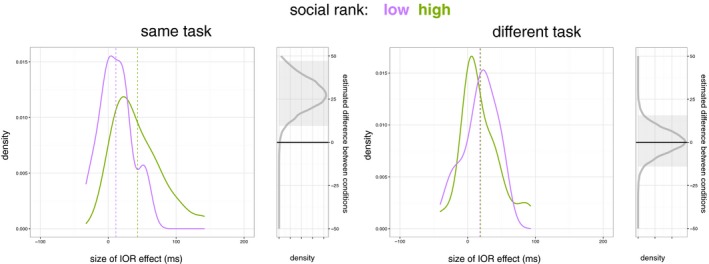
Purple and green lines show the distribution of inhibition of return effects in each social rank condition, across the two task conditions with dashed lines indicating the means for each social rank condition. On the right of each task condition, the gray line shows the Bayesian posterior probability distributions for the difference between conditions, and the gray area shows the 95% credibility interval (HDI) for this difference.

First, results from the anova indicated a significant effect of Target Location. Participants were generally slower to respond to cued locations (*M = *466 ms, *SEM = *15 ms) than uncued locations (*M = *443 ms, *SEM = *14 ms), corresponding to an overall IOR effect of about 23 ms*, F*(1, 43)* = *59.96, *p *<* *.001, $\eta_{p}^{2}$ = .582. We also observed a significant two‐way interaction between Partner Rank and Target Location, *F*(1, 43) = 7.78, *p = *.008, $\eta_{p}^{2}$ = .153. Importantly, this two‐way interaction was further qualified by Task Type, as indicated by a significant three‐way interaction between Partner Rank, Target Location and Task Type, *F*(1, 43) = 5.64, *p = *.022, $\eta_{p}^{2}$ = .116. To further interpret this three‐way‐interaction, we investigated participants' IOR effects as a function of their partners' social rank separately when they believed they were engaged in the same or a different task. Fig. 3 plots the size of the IOR effects for each of the experimental conditions, with 95% credibility intervals for the Bayesian estimation of the differences between high‐ and low‐status conditions.

When engaged in the same task as the interaction partner, the social rank of the interaction partner *moderated* IOR effects, as indicated by a significant two‐way interaction between Partner Rank and Target Location, *F*(1, 43) = 11.84, *p = *.001, $\eta_{p}^{2}$ = .216. When interacting with a higher ranked interaction partner, participants were significantly slower to respond to cued locations (*M = *469 ms, *SEM* = 23 ms) than uncued locations (*M = *426 ms, *SEM* = 21 ms), *p *<* *.001. However, when interacting with a lower ranked interaction partner, this was less the case (*M*_cued_ = 472 ms, *SEM*_cued_ = 24 ms; and *M*_uncued_ = 460 ms, *SEM*_uncued_ = 23 ms, respectively; *p = *.045). IOR effects amounted to *M = *43 ms (*SEM* = 7 ms) when the interaction partner was higher in social rank, compared to *M = *11 ms (*SEM* = 5 ms) when the interaction partner was lower in social rank. An independent‐samples *t* test indicated that this difference was highly significant, *t*(40.37) = 3.53, *p = *.001, *d = *1.11 (correcting degrees of freedom to account for unequal variance between groups).

In contrast, when engaged in a different task as the interaction partner, the social rank of the interaction partner *did not moderate* IOR effects, *F*(1, 43) < 1, *p = *.904, $\eta_{p}^{2}$ = .000. When interacting with a higher ranked interaction partner, participants were slower to respond to cued locations (*M = *446 ms, *SEM* = 19 ms) than uncued locations (*M = *427 ms, *SEM* = 18 ms), *p = *.005. And this was also the case when interacting with a lower ranked interaction partner, (*M*_cued_ = 479 ms, *SEM*_cued_ = 21 ms; and *M*_uncued_ = 461 ms, *SEM*_uncued_ = 19 ms, respectively; *p = *.003). IOR effects were of similar magnitude, *t*(43) < 1, *p = *.904, *d = *.04, when the interaction partner was higher in social rank, *M = *19 ms (*SEM* = 6 ms), compared to when the interaction partner was lower in social rank, *M = *18 ms (*SEM* = 5 ms).

Another way to interpret the observed three‐way interaction is to assess the effect of being engaged in the same or a different task for higher and lower ranked interaction partner separately. We found that for higher ranked interaction partner, task type *moderated* IOR effects, as indicated by a significant target location by task type interaction, *F*(1, 23) = 6.13, *p* = .021, $\eta_{p}^{2}$ = .210. Thus, IOR effects were almost twice as large when being engaged in the same task as the higher ranked interaction partner (*M = *43 ms; *SEM* = 7 ms), compared to when engaged in a different task as the higher ranked interaction partner (*M = *19 ms; *SEM* = 5 ms), *t*(23) = 2.48, *p = *.021. In stark contrast, for lower ranked interaction partners, task type *did not moderate* IOR effects, *F*(1, 20) < 1, *p = *.426, $\eta_{p}^{2}$ = .032. Here, IOR effects were of similar magnitude when being engaged in the same task as the lower ranked interaction partner (*M = *11 ms; *SEM* = 5 ms), compared to when being engaged in a different task as the lower ranked interaction partner (*M* = 18 ms; *SEM* = 5 ms)*, t*(20) < 1, *p* = .426.

Consistent with the frequentist inference, and as shown in Fig. 3, the distribution of effects and credibility intervals for the differences between partner rank conditions provide strong evidence for a non‐zero difference between the IOR effects in the same task but not in the different task condition: When participants believed they were engaged in the same task as the interaction partner, the 95% Bayesian credibility interval, shown by the gray box, was from 9.5 to 47.1, demonstrating good evidence for a non‐zero difference between the IOR effects of higher and lower ranking conditions. In contrast, when engaged in a different task as the interaction partner, the 95% Bayesian credibility intervals included zero (95% CI: −14.1 to 15.7), showing no difference between the IOR effects as a function of partner rank condition.

#### 3.2.3 Comparing results from Experiments 1 and 2

If manipulating the *beliefs* about the social relevance of non‐social cues alone can modulate the magnitude of spatial orienting effects, whether it is because the cue is connected to the interaction partner as in Experiment 1, or whether it is because participants interacted with a higher ranked interaction partner on the same task as in Experiment 2, the absence of social relevance should yield comparable spatial orienting effects, as they reflect the non‐social nature of the stimuli when being responded to in the presence of another person. Therefore, we reasoned that spatial orienting effects in trials where participants believed eye trackers to be disconnected in Experiment 1 and in trials where participants believed they were working on a different task as their interaction partner in Experiment 2, the spatial orienting effects should be comparable. To test this prediction, we compared the computer‐generated cue trials from Experiment 1 and the different task trials from Experiment 2 in a 2 (Experiment: Exp 1 vs. Exp 2) × 2 (Target Location: cued vs. uncued) mixed‐model anova with the first factor between subjects and the latter factor within subjects.

We observed that participants responded with an approximately similar speed in Experiment 1 (*M = *425 ms, *SEM* = 12 ms) and Experiment 2 (*M = *452 ms, *SEM* = 12 ms), *F*(1, 92) = 2.49, *p = *.118, $\eta_{p}^{2}$ = .03. Moreover, we observed a significant main effect of Target Location, with cued trials (*M = *446 ms, *SEM* = 9 ms) slower than uncued trials (*M = *431 ms, *SEM* = 8 ms), consistent with an overall IOR effect of about 15 ms, *F*(1, 92) = 31.05, *p *<* *.001, $\eta_{p}^{2}$ = .25. Importantly, this effect was comparable in both experiments, as we did not observe a significant interaction between Experiment and Target Location, *F*(1, 92) = 1.51, *p = *.22, $\eta_{p}^{2}$ = .02. Thus, in the absence of any social relevance of the cue, either because the cue was computer‐generated or because the interaction partner was engaged in a different task, we observed spatial orienting of roughly the same magnitude.

### 3.3 Discussion

In Experiment 2, we found that participants took significantly longer to respond to spatial locations that they believed a higher ranked interaction partner had just looked at, but not to spatial locations that they believed a lower ranked interaction partner had just looked at. Importantly, this was only the case when being engaged in the same task but not when being engaged in a different task as the interaction partner. Experiment 2 also showed that when interacting with a lower ranked interaction partner, IOR effects remained similar in magnitude independent of task type, as if the lower ranked interaction partner was irrelevant to the task at hand. In contrast, when interacting with a higher ranked interaction partner, the magnitude of IOR effects doubled in size when being engaged in the same task.

Simply being in the presence of the higher or lower ranked interaction partner who happened to be looking at the same stimuli at the same time was insufficient to modulate interpersonal spatial orienting. In fact, the magnitude of the spatial orienting effects in this condition was roughly the same as when the cue was computer‐generated in Experiment 1. Thus, only if participants were made to believe that they shared the same goals and intentions as the interaction partner did the specifics of who the interaction partner was then matter for the modulation of interpersonal spatial orienting. This finding is inconsistent with the *attentional state hypothesis*, and it provides evidence in support of the *intentional state hypothesis*. Therein, Experiment 2 adds additional support for the idea that beliefs about the social relevance of a non‐social cue modulate interpersonal spatial orienting effects.

## 4 General discussion

Beliefs about the social relevance of a non‐social cue influence interpersonal spatial orienting, independent of the social appearance of the cue. Specifically, our research yielded three important findings: First, Experiment 1 showed that a non‐social red dot representing the gaze location of an interaction partner amplified spatial cue‐target compatibility effects compared to when the cue was computer‐generated.

Second, Experiment 2 showed that this effect was further modulated by beliefs about who the interaction partner was and what the interaction partner was doing. Only when participants believed that they were doing the same task as their interaction partner, and thus the latter became relevant to the participant, did higher compared to lower ranked interaction partner yield stronger interpersonal spatial cue‐target compatibility effects.

Third, Experiment 2 in combination with results from Experiment 1 also showed that the mere presence of another person did not suffice to change spatial orienting. Indeed, results from Experiment 2 rule out the possibility that merely being able to see where the interaction partner is looking on their screen would be sufficient to modulate spatial attention. Attributing attentional states without also attributing intentional states did not change interpersonal spatial orienting effects in our studies.

Taken together, we show that the social relevance of a non‐social cue, representing who another person is and what this other person is doing, modulates interpersonal spatial cue‐target compatibility effects. Importantly, in all our experiments we employed identical non‐social stimuli, and the only thing we manipulated were participants' beliefs about the social context.

Our results make two important contributions to our understanding of how visual attention unfolds within social contexts. They suggest that social cues can have a stronger effect upon spatial orienting than non‐social cues. They also show that these differences in orienting effects do not depend on *social appearance* of the cue alone, but they can be the result of *social beliefs* instead. In this way, a red dot that looks like any other non‐social stimulus can have a more significant influence on visual attention when participants believe that it is connected to another person. We believe this is strong evidence for the “*social*” nature of the interpersonal spatial orienting effect debated in the literature.

Our findings also have important implications for research on human memory and social learning. Initial evidence from the joint attention literature suggests that stimuli that are attended to along with another person are faster and more accurately identified in signal detection tasks (Shteynberg, 2010), and they are more likely to be recalled subsequently (Eskenazi, Doerrfeld, Logan, & Knoblich, 2013). Moreover, under conditions of joint attention, participants are also more likely to mimic the behavior of others, one important form of social learning (Shteynberg & Apfelbaum, 2013). Our research can add to this literature suggesting that the social relevance of another person, who this person is and what this person is doing, might further moderate the effect of joint attention on human memory and social learning.

### 4.1 The fourth wall of cognitive science

The attentional focus of another person can act as a strong cue for our own attention, as elegantly demonstrated in the gaze cueing literature (e.g., Friesen & Kingstone, 1998; Frischen et al., 2007). Someone else's eye and hand movements can even trigger our own IOR mechanisms (Skarratt et al., 2010; Welsh et al., 2005). But in all of these experimental demonstrations, the social cue is *visibly* social: a face, a hand, or a head turn. And in each case, researchers have shown that the attentional effect of a social cue is roughly equivalent to a non‐social cue such as an arrow. Here, we showed for the first time that minimal changes of participants' beliefs about the social nature of a non‐social cue suffice to increase social over non‐social spatial orienting effects.

Our findings highlight the importance of social beliefs on visual attention converging with similar results from the gaze cueing literature. For example, gaze cueing effects are modulated by participants' beliefs about the person cueing their attention (Dalmaso et al., 2012). Another study found that attributing intentional states to non‐social robots, when participants believed that robots were controlled by human agents, mirrored the cueing effects seen when participants viewed the face of another human being (Wiese et al., 2012). Differences in beliefs can even shape habitual ways of attending to others, as demonstrated by recent research that demonstrated cultural differences in gaze cueing (Cohen, Sasaki, German, & Kim, 2015).

Our results converge with other recent theory and research making this same point: Social context has a pervasive effect on visual attention (Richardson & Gobel, 2015). For example, research has repeatedly shown that visual attention measured in the laboratory can differ from visual attention as it is employed in the real world (Kuhn, Teszka, Tenaw, & Kingstone, 2016; Risko, Laidlaw, Freeth, Foulsham, & Kingstone, 2012; Risko et al., 2016). When people look at others' faces, gaze patterns vary significantly depending on when looking at a live or pre‐recorded video (Laidlaw, Foulsham, Kuhn, & Kingstone, 2011), or whether the face is looking directly at them or not (Gobel, Chen, & Richardson, 2017). Indeed, people are very much aware of social scripts that govern when it is appropriate to look and when it is not (Foulsham, Walker, & Kingstone, 2011; Laidlaw, Rothwell, & Kingstone, 2016; Wu, Bischof, & Kingstone, 2013).

For example, Gobel, Kim, and Richardson (2015) had participants look at videos of faces starring into the camera, while they themselves were being videotaped. For half of the participants, the people in the videoclips were described as higher in social rank, whereas for the other half of participants they were described as lower in social rank. Further, in half the trials, participants were told that the people in the videoclips were previous participants who would return to the lab in order to view their video recordings—creating the illusion of the faces on screen looking back at participants. In half the trials, participants were told that the video recordings would not be reviewed—creating the illusion of them looking at the faces without the latter looking back. Results showed that participants looked longer to the eyes of higher than lower ranked target faces, but only if the faces were believed to not look back at them (Gobel et al., 2015). This suggests that when looking directly at someone, the effects of social rank on visual attention also depend upon higher order beliefs about the social situation.

### 4.2 Limitations and future research

We will end with a note of caution. The underlying mechanisms of IOR are still debated (Dukewich & Klein, 2015; Klein, 2000). While some researchers have argued that IOR affects information processing at earlier attentional stages (e.g., Posner et al., 1985; Prime & Ward, 2004), others have argued that IOR affects information processing at later stages, when decisions are selected (e.g., Prinzmetal, Taylor, Myers, & Nguyen‐Espino, 2011; Taylor & Klein, 1998). While it is beyond the scope of the current research, future studies could use our current methodology along with neuroscientific measures to pinpoint the stages of information processing that are influenced by IOR, as well as the underlying mechanisms of such socially tuned IOR effects.

One possibility is that top–down processes, such as volitional control or stimulus‐associated reward (e.g., Bucker & Theeuwes, 2014; Lupiáñez, Weaver, Tipper, Madrid, & Castillo, 2001; Tipper & Kingstone, 2005), might explain our findings. For example, associating a stimulus with a previously learned reward captures attention (Anderson, Laurent, & Yantis, 2011), and it improves target discrimination (Maclean & Giesbrecht, 2015). Importantly, in our study, we found that higher compared to lower ranked interaction partners only had a differential effect on interpersonal spatial orienting, when being task relevant. This may suggest that the social relevance of a cue operates at least in part differently to its monetary reward. It remains an intriguing question for future research whether the social relevance of a cue or the monetary value of a cue relies on the same or different mental representations and neural circuitry.

Another possibility is that changes in arousal might account for our findings. For example, the mere presence of another person reduces Stroop interference effects (Huguet, Galvaing, Monteil, & Dumas, 1999), and social comparison with a better performing co‐actor improves performance on illusionary conjunctions (Muller, Atzeni, & Butera, 2004). One explanation for these findings is that completing a task with another person increases arousal, which leads to more cognitive resources being consumed, and as a result reduces attentional focus to peripheral stimuli (Muller & Butera, 2007; Normand, Bouquet, & Croizet, 2014). Although it is difficult to imagine how such a mechanism would result in larger IOR effects in the present set of experiments, increased levels of arousal as one possible mechanism for why the social relevance of a cue influences spatial orienting remains an interesting avenue for future studies.

One important limitation of our experiments is that we presented 25% cued compared to 75% uncued trials. Previous research has shown that under these circumstances, participants are able to learn that targets are less likely to occur in cued compared to uncued spatial locations (e.g., Friesen, Ristic, & Kingstone, 2004). As a consequence, they may have been slower to respond to cued compared to uncued spatial locations, because they develop counter‐predictive expectations over time. However, recent research using a 50% predictive cue replicated the findings reported here (Gobel, Bullock, Kim, Richardson, & Giesbrecht, unpublished data). While we acknowledge that our data do not allow us to rule out this possibility, we do believe that it is unlikely that the predictability of the cue is the only mechanism explaining our findings. All participants saw exactly the same non‐social cues, and the only thing that we manipulated across experimental conditions was the participant's belief about the social relevance of the cue.

Another important question that our research raises is whether the effect of social relevance on spatial orienting is limited to volitional eye movements or whether it can also occur within more reflexive spatial orienting. Previous research differentiates between two attentional systems implicated in IOR. While one system orients attention to the cued location, the other disengages and reorients attention away from the cued location to the behaviorally relevant stimulus (Corbetta & Shulman, 2002). Indeed, it seems that these systems rely on distinct functional networks (Kincade, Abrams, Astafiev, Shulman, & Corbetta, 2005; Thiel, Zilles, & Fink, 2004). A recent study found that monetary reward modulated later stage disengaging and reorienting aspects of spatial attention, but not the early occurring orienting aspects (Bucker & Theeuwes, 2014). Beyond the scope of the present research, it would be fascinating to observe that the social relevance of a cue influences spatial orienting as early as 50–100 ms after stimulus onset. At the same time, if social representations fail to impact these early occurring aspects of spatial orienting, this would illustrate an important boundary condition to their effect. It remains an intriguing question for future research to describe the time course and specific mechanisms underlying the here observed social relevance effects.

Finally, the present results demonstrate that minimal social contexts influence one aspect of visual attention: spatial orienting. Future research is needed to test the influence that the social relevance of a cue might yield onto other attentional processes than IOR. In the present research, we made a first contribution to the attentional literature showing that one basic attentional process—spatial orienting—is modulated by beliefs about the social relevance of the cue—whether the cue is connected to another person, who this person is, and what this person is doing.

## 5 Conclusion

People actively follow the attentional focus of others. Our results reveal a new aspect of employing visual attention in social situations. Even minimal and artificial social contexts created in a laboratory—manipulating participants' beliefs about a non‐social cue representing another person's gaze—were enough to influence spatial orienting of the participant. This basic process of visual attention was further modulated by beliefs about the social relevance of the other person such as its social rank and its intentional state. Visual attention is not only guided by the physical salience of one's environment but also by the mental representation of its social relevance.
